# McGISH identification and phenotypic description of leaf rust and yellow rust resistant partial amphiploids originating from a wheat × *Thinopyrum* synthetic hybrid cross

**DOI:** 10.1007/s13353-016-0343-8

**Published:** 2016-02-27

**Authors:** Klaudia Kruppa, Edina Türkösi, Marianna Mayer, Viola Tóth, Gyula Vida, Éva Szakács, Márta Molnár-Láng

**Affiliations:** Agricultural Institute, Centre for Agricultural Research, Hungarian Academy of Sciences, H-2462 Martonvásár, Brunszvik u. 2 Hungary

**Keywords:** FISH, Leaf rust resistance, Multicolour GISH, Partial amphiploid, *Thinopyrum intermedium × Thinopyrum ponticum* synthetic hybrid (Agropyron glael)

## Abstract

A *Thinopyrum intermedium* × *Thinopyrum ponticum* synthetic hybrid wheatgrass is an excellent source of leaf and stem rust resistance produced by N.V.Tsitsin. Wheat line Mv9kr1 was crossed with this hybrid (Agropyron glael) in Hungary in order to transfer its advantageous agronomic traits into wheat. As the wheat parent was susceptible to leaf rust, the transfer of resistance was easily recognizable in the progenies. Three different partial amphiploid lines with leaf rust resistance were selected from the wheat/*Thinopyrum* hybrid derivatives by multicolour genomic in situ hybridization. Chromosome counting on the partial amphiploids revealed 58 chromosomes (18 wheatgrass) in line 194, 56 (14 wheatgrass) in line 195 and 54 (12 wheatgrass) in line 196. The wheat chromosomes present in these lines were identified and the wheatgrass chromosomes were characterized by fluorescence in situ hybridization using the repetitive DNA probes Afa-family, pSc119.2 and pTa71. The 3D wheat chromosome was missing from the lines. Molecular marker analysis showed the presence of the *Lr24* leaf rust resistance gene in lines 195 and 196. The morphological traits were evaluated in the field during two consecutive seasons in two different locations.

## Introduction

The perennial wheatgrasses possess several favourable features for wheat improvement, such as tolerance to biotic and abiotic stresses, leading to better crop safety, yield and quality. Intermediate wheatgrass [*Thinopyrum intermedium* (Host) Barkworth & D.R. Dewey] and tall wheatgrass [*Thinopyrum ponticum* (Podp.) Z.-W. Liu & R.-C. Wang] are the two most common introduced species. Because of the sterility of wheat × *Thinopyrum* F_1_ hybrids, complete amphiploids or more frequently partial amphiploids are the starting material for successful gene transfer (Jiang et al. 1994). Colchicine treatment on the F_1_ hybrids leads to the formation of chromosome-doubled amphiploid plants. Partial amphiploids can be selected among the progenies of backcrossed F_1_ hybrids. The high number of homoeologous chromosomes causes genetic instability in the amphiploids. As a result of substitutions and deletions, partial amphiploid plants carry a stabilized genome. In the case of bread wheat/polyploid *Thinopyrum* partial amphiploids, genetically stable lines with 56 chromosomes (8×) were reported (Banks et al. 1993; Fedak et al. 2000; Han et al. 2004; Oliver et al. 2006; Sepsi et al. 2008; Bao et al. 2009; Chang et al. 2010; Georgieva et al. 2011; and Zheng et al. 2014), while in durum wheat/polyploid *Thinopyrum* partial amphiploids 42 chromosomes (6×) were observed (Zeng et al. 2013).

Intermediate and tall wheatgrasses are not only important forage crops but also valuable gene reservoirs for wheat (*Triticum aestivum* L.) improvement. Almost half of leaf rust resistance genes, 30 % of stem rust resistance genes and 10 % of yellow rust resistance genes have been introduced into bread wheat from closely related and/or wild species (Salina et al. 2015). A significant proportion of them were derived from polyploid *Thinopyrum* species (Wang 2011). Chromosomal segments of *Thinopyrum ponticum* (2n = 10× = 70) carrying the leaf rust resistance genes *Lr19* (Friebe et al. 1994), *Lr24* (McIntosh et al. 1977) and *Lr29* (Procunier et al. 1995) and the stem rust resistance genes *Sr24* (Sears 1973), *Sr25* (McIntosh et al. 1977), *Sr26* (Friebe et al. 1994) and *Sr43* (Kim et al. 1993) were transferred into wheat. *Lr24* is completely linked with *Sr24* while *Sr25* often shows complete linkage to *Lr19. Thinopyrum intermedium* was used as a source of the *Lr38* (Friebe et al. 1992), *Sr44* (Friebe et al. 1996), *Bdv2* (Banks et al. 1995), *Bdv3* (Sharma et al. 1995), *Bdv4* (Lin et al. 2006), *Yr50* (Liu et al. 2013) and *Wsm1* (Liang et al. 1979) resistance genes via wheat-alien introgressions. These translocations can result from either spontaneous or induced (Friebe et al. 1996) recombination.

Generic relationships within the *Triticeae* are problematic (Kellogg 2006). Tall wheatgrass was previously classified as *Agropyron elongatum* and intermediate wheatgrass as *Agropyron glaucum* in the genus *Agropyron* (Hitchcock 1951). Dewey (1984) reduced the *Agropyron* genus based on the presence of the P genome. Tall and intermediate wheatgrass were relocated to the *Thinopyrum* genus as *Thinopyrum ponticum* (Podp.) Z.-W. Liu & R.-C. Wang and *Th. intermedium* (Host) Barkworth & D.R. Dewey, respectively. Polyploid *Thinopyrum* species contain genomes similar to the J (J^b^, E^b^) genome of the diploid *Th. bessarabicum* (Östergren 1940) or the E (J^e^, E^e^) genome of *Th. elongatum* (Cauderon and Saigne 1961), which are closely related (Ceoloni et al. 2014), and sometimes a third genome from *Pseudoroegneria* designated as St, previously designated as S (Wang et al. 1995).

The first successful crosses between wheat and wheatgrasses were made in 1930 by NV Tsitsin (Armstrong 1936). Wheat × wheatgrass hybrids were produced to breed wheat with perennial growth habit in the former Soviet Union (Verushkine and Shechurdine 1933). A synthetic hybrid was produced by crossing *Th. intermedium* (former name *Agropyron glaucum*) with *Th. ponticum* (former name *Agropyron elongatum*) in the 1950s by NV Tsitsin, the aim being to analyse the genome composition of the *Agropyron* species used in wheat/wheatgrass crosses (Tsitsin 1979). The hybrid plants were named as Agropyron glael by Tsitsin, as an abbreviation of *glaucum* and *elongatum*. This name (A. glael) will be used hereafter in this article. The hybrid plants had 56 chromosomes. A number of *A. glael* plants were maintained in Martonvásár (Hungary) thanks to cooperation between the Hungarian Academy of Sciences and the Moscow Research Institute of Agriculture “Nemchinovka” in the 1960s.

The aim of this study was to describe the chromosome composition of three newly selected wheat/A. glael partial amphiploids by means of multicolour genomic in situ hybridization (mcGISH) and fluorescence in situ hybridization (FISH). A further aim was to characterize artificial powdery mildew inoculation and spontaneous leaf rust and yellow rust infection together with the molecular marker analysis of some *Thinopyrum*-derived *Lr* genes present in the lines. The morphological parameters of the partial amphiploid lines were also described.

## Materials and methods

### Plant material

The A. glael perennial wheatgrass clone was kindly provided by GD Lapchenko from the Moscow Research Institute of Agriculture ’Nemchinovka’. The clone has been maintained in the perennial nursery in Martonvásár since the 1960s by the Hungarian breeder Dezső Szalay. Wheat genotype Mv9kr1, containing both the recessive crossability alleles (*kr1kr1kr2kr2*) (Molnár-Láng et al. 1996), was crossed with A. glael in 2001. Young inflorescences of F_1_ plants were used for callus induction and were multiplied in tissue culture as described by Molnár-Láng et al. (1991). Regenerated plants were grown in the phytotron under the conditions described by Tischner et al. (1997). Chinese Spring wheat was the pollinator during backcrossing. The BC_1_F_5_-BC_1_F_8_ lines, were analysed cytogenetically.

### Sequential mcGISH and FISH

Chromosome preparation was carried out as described by Lukaszewski et al. (2004). McGISH was performed in order to simultaneously visualize the different *Thinopyrum* chromosomes in the BC_1_ self-pollinated progenies. J (E^b^) genomic DNA from *Th. bessarabicum* labelled with biotin-11-dUTP (Roche Diagnostics, Mannheim, Germany) and St genomic DNA from *Ps. spicata* labelled with digoxigenin-11-dUTP was produced using the random primed labelling protocol. The hybridization mixture contained 100 ng each of the labelled probes/slide, dissolved in a 15 μl mixture of 100 % formamide, 20 × SSC and 10 % dextran-sulphate at a ratio of 5:1:4, and 3000 ng *Triticum aestivum* DNA (BBAADD) as a block. Hybridization was performed at 42 °C overnight. Streptavidin-FITC (Roche) and Anti-Digoxigenin-Rhodamine (Roche) dissolved in TNB (Tris-NaCl-blocking buffer) were used in the detection phase. After rinsing off the mcGISH signals, three-colour FISH was performed using three repetitive DNA probes: Afa-family, pSc119.2 and pTa71. Hybridization and detection were carried out as reported by Kruppa et al. (2013). The slides were screened using a Zeiss Axioskop-2 fluorescence microscope equipped with filter sets appropriate for DAPI (Zeiss Filterset 01), and for the simultaneous detection of FITC and Rhodamine (Zeiss filter set 24). Images were captured with a Spot CCD camera (Diagnostic Instruments) and processed with Image Pro Plus software (Media Cybernetics).

### Molecular marker analysis

Four primer pairs were used for the detection of the absence or presence of certain *Thinopyrum*-derived leaf rust and stem rust resistance genes in the partial amphiploid lines. Genomic DNA was extracted from fresh young leaves of wheat cultivars Chinese Spring, Mv9kr1, the wheatgrass species *Th. intermedium, Th. ponticum*, the synthetic hybrid A. glael, the positive control wheat lines SO91-1027 (*Lr19*), TC24 (Thatcher*6/Agent, *Lr24*), TC29 (Thatcher*6//CS7D/Ag#11, *Lr29*) and Sunelg (*Sr26*) and the three partial amphiploid lines (lines 194, 195, 196) with a DNeasy Plant Kit (Qiagen, Germany). The STS marker *STSLr19*_*130*_ with the primer pair GbF-GbR (*Lr19*, Prins et al. 2001), STS marker *J09-STS* with the primer pair J09/1-J09/2 (*Lr24*, Schachermayr et al. 1995) and a SCAR marker with Lr29F18-Lr29R18 primers (*Lr29*, Procunier, http://maswheat.ucdavis.edu/protocols/Lr29/), were used to reveal the presence of the *Lr19*, *Lr24* and *Lr29* leaf rust resistance genes (derived from *Thinpyrum* sp.) in the partial amphiploid lines. Multiplex PCR with markers *Sr26#43* (a dominant STS marker for the presence of *Sr26*) and BE518379 (6AL-specific, dominant for the absence of *Sr26*) (Liu et al. 2010) were used to characterize the presence of *Sr26*. PCR reactions were performed in an Applied Biosystem 9700 PCR (Life Technologies, California, USA) in a final volume of 20 μl containing 200 ng DNA template, 5× Green Go Taq Flexi Buffer (Promega), 2.34 mM MgCl_2,_ 0.9 μM of each dNTP, 10 pmol forward and reverse primers and 1 U GoTaq DNA Polymerase (5 U/μ, Promega). The PCR products were separated using SeaKem 1.5 % agarose gels (Lonza, Rockland, ME, USA) and the fragments were stained using ethidium bromide. A 100-bp DNA ladder (GelPilot 100 bp Plus Ladder, Qiagen, Germany) was used to estimate molecular weight. The patterns were documented and analysed using a Syngene G-BOX documentation system (Syngene, Maryland, USA).

### Phenotypic evaluation of the plants

The partial amphiploid lines and the parental wheat genotype (Mv9kr1) were grown in the pesticide-free Tükrös nursery in Martonvásár in two consecutive seasons (2013–2014 and 2014–2015) with 10 seeds in each 1 m row and a row distance of 15 cm. The same genotypes were sown in the breeder’s nursery in Lászlópuszta in the 2014–2015 season in plots of 2 m^2^. Ten plants were randomly selected from each genotype for analysis. Plant height and tillering (spikes per plant) were measured in the field immediately before harvest. The traits fertility (seeds per spikelet), length of the main spike, number of spikelets per main spike and number of seeds per main spike were measured after harvest. Differences in morphological characteristics between the partial amphiploid line and the control Mv9kr1 genotype were determined by means of the MS Excel Student’s t-test for paired data at the P = 0.05 significance level.

### Artificial powdery mildew inoculation and spontaneous leaf rust and stripe rust infection

Powdery mildew resistance was tested under greenhouse conditions. *Blumeria graminis f.sp. tritici* isolate P07-14 (virulent on differentials with genes *Pm1, Pm2, Pm3a, Pm3d, Pm4a, Pm4b, Pm5, Pm6, Pm7, Pm8 or Pm17* or the gene combinations *Pm1,2,9*, *Pm2,4b,8, Pm2,6, Pm2,Mld*; avirulent on: *Pm3b, Pm3c, Pm3f*) was used for inoculation. Ten plants of each genotype (2 partial amphiploids + 2 parents + Carsten V susceptible check) were grown in three randomized replications under an isolator (18 °C, relative air humidity of 80–90 %). The inoculum was shaken on to the leaf surface 9–10 days after sowing. The type of infection was determined ten days after inoculation using the method recommended by Nover (1957). Resistant genotypes gave a score of 0–2, while those with scores of 3–4 were susceptible.

Each year several rows of the leaf rust (*Puccinia triticina*) -susceptible wheat cultivar Mv9kr1 were planted in the nursery adjacent to the plots of Mv9kr1/A. glael BC_1_ selfed progenies. Leaf rust and yellow rust (*Puccinia striiformis* f.sp. *tritici*) resistance were described using observations on spontaneous infection in the last three years.

## Results

### Crosses

The hybridization of Mv9kr1 wheat and A. glael resulted in 255 F_1_ grains. The first successful backcrossing with the wheat genotype Chinese Spring resulted in five BC_1_ grains in 2004, but only two of them were viable. The first BC_1_ plant (line 0566) carried 49 chromosomes and was backcrossed with Mv9kr1, but none of the 11 BC_2_ grains originating from 0566 were viable. The other BC_1_ plant (No.0567, 62 chromosomes) had four spikes, three of which were self-pollinated resulting in 46 BC_1_F_2_ grains, while the fourth was backcrossed with Mv9kr1, resulting in 19 BC_2_ seeds. Derivatives of these plants have been maintained, self-pollinated and selected for leaf rust resistance in the Tükrös nursery since 2006. Plants of the leaf rust-resistant BC_1_F_5_-BC_1_F_8_ lines were analysed cytogenetically and grown in the phytotron.

### Molecular cytogenetic analysis

#### Partial amphiploid line 194: 58 chromosomes

The chromosome number and genome composition of the wheat–A. glael partial amphiploids were analysed in somatic metaphase spreads from 5–20 individual plants by sequential mcGISH and FISH.

**Fig. 1:**
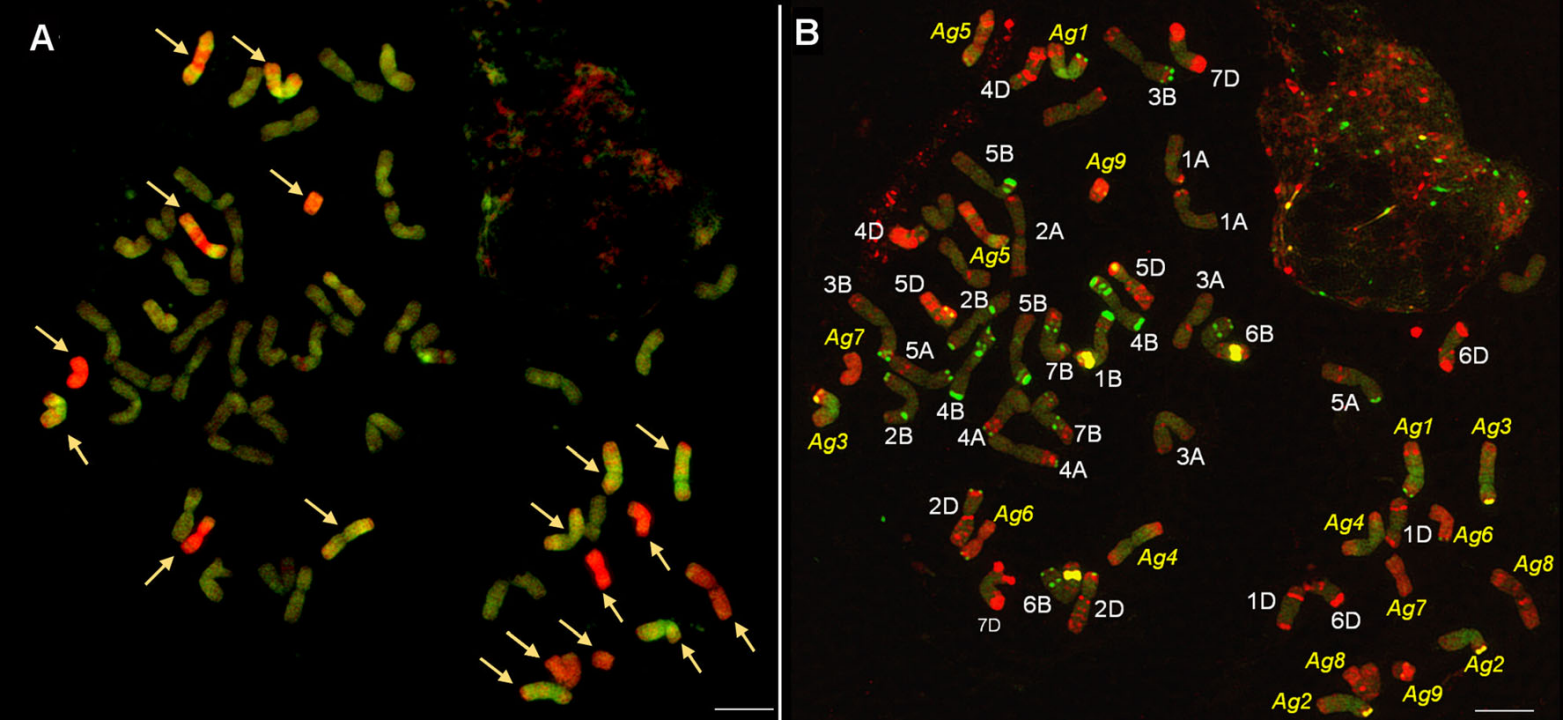
**a** Multicolour genomic in situ hybridization (mcGISH) on mitotic chromosomes of the partial amphiploid lines 194 derived from the Mv9kr1 (wheat) × *Thinopyrum* synthetic hybrid (Agropyron glael, hybrid of *Thinopyrum intermedium* and *Thinopyrum ponticum*) cross using J (*Thinopyrum bessarabicum*, green) and St (*Pseudoroegneria spicata*, red) genomic DNA probes. Wheat chromosomes are unlabelled (brown). Alien chromosomes are indicated with arrowheads. **b** The fluorescent in situ hybridization (FISH) pattern on the same cell of lines 194 using Afa-family (red), pSc119.2 (green) and pTa71 (yellow) repetitive DNA probes. A. glael chromosomes are numbered in yellow, not based on homology, while the wheat chromosomes are numbered in white. Scale bar: 10 μm

McGISH allowed nine pairs of A. glael chromosomes to be discriminated (Fig. 1a). Biotin-labelled J genomic DNA hybridized to the entire length of four pairs of submetacentric chromosomes (Ag1-Ag4). Ag5 exhibited a special hybridization pattern: St genomic DNA hybridized to the centromeric and pericentromeric region, while J genomic DNA hybridized to the other parts of the chromosome with the exception of the telomeric region, which remained unlabelled. This chromosome could be identified as J^S^. The remaining four pairs of chromosomes were labelled by St genomic DNA (Ag6-Ag9) but with faint intensity in the case of Ag8. Chromosomes belonging to the St genome differed greatly in chromosome length and fluorescence intensity. The smallest St chromosome was acro- or telocentric (Ag9), while the others were nearly metacentric. Among the 18 fluorescing chromosomes, two pairs carried a terminal unlabelled region, suggesting that intergenomic rearrangement had taken place. St genomic DNA gave a strong hybridization signal on the satellite region of the wheat chromosomes. J genomic DNA hybridized, though with lower intensity, to six wheat chromosomes, while others were unlabelled.

**Fig. 2:**
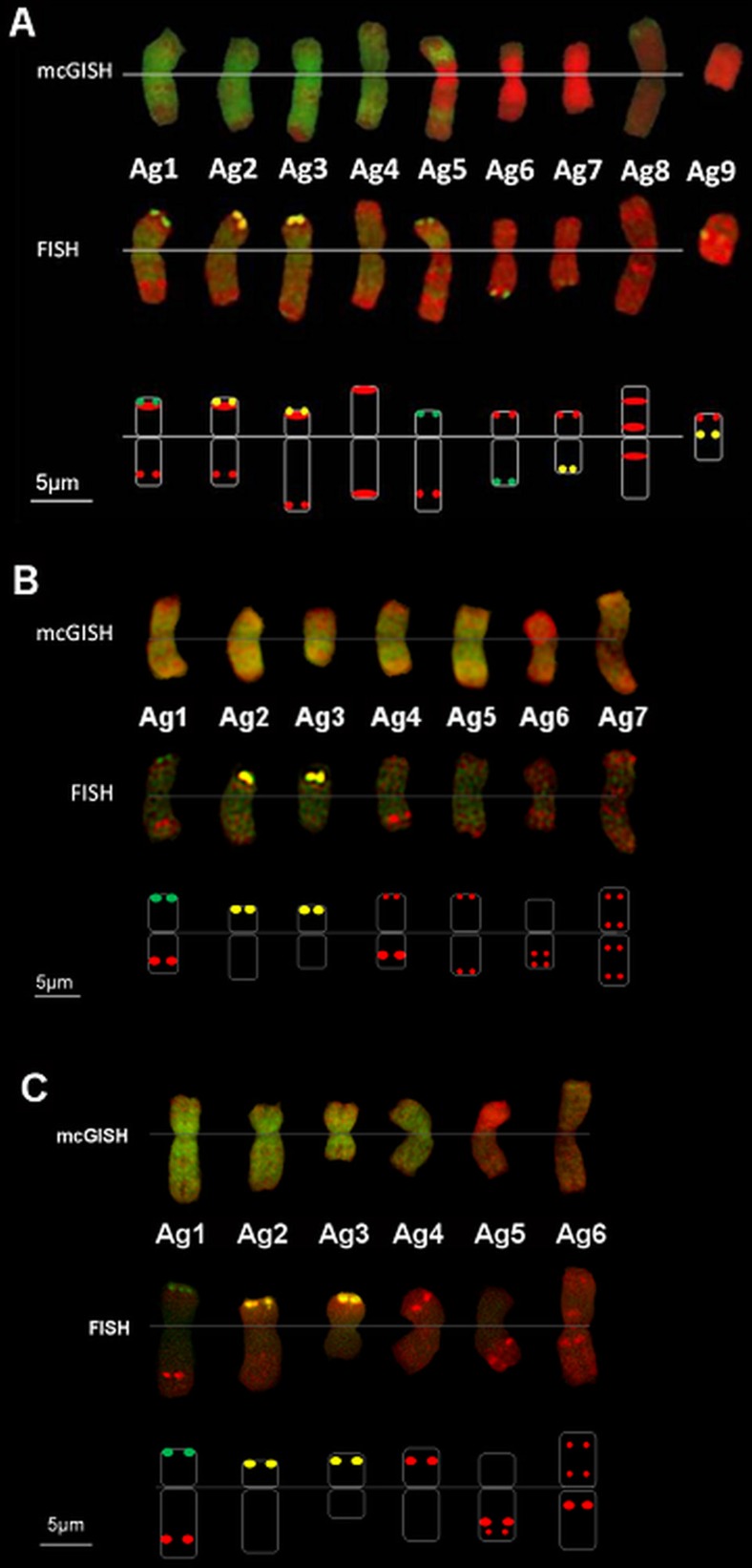
Wheatgrass chromosomes in Mv9kr1 wheat/*Thinopyrum* synthetic hybrid (Agropyron glael, hybrid of *Thinopyrum intermedium* and *Thinopyrum ponticum*) partial amphiploid lines 194 (**a**), 195 (**b**) and 196 (**c**). Multicolour genomic in situ hybridization (mcGISH) karyograms using J (*Thinopyrum bessarabicum*, green) and St (*Pseudoroegneria spicata*, red) genomic DNA are presented in the top lanes. Fluorescent in situ hybridization (FISH) karyograms resulting from simultaneous hybridization with Afa-family (red), pSc119.2 (green) and pTa71 (yellow) repetitive DNA probes are presented in the middle lanes. Idiograms of the FISH patterns of the wheatgrass chromosomes are shown in the bottom lanes. Scale bar: 5 μm

Twenty pairs of chromosome were blocked by wheat DNA instead of 21, showing that one pair of wheat chromosomes was substituted by a pair of alien chromosomes. FISH with repetitive DNA probes (Afa-family, pTa71, pSc119.2) was used for the identification of the 40 wheat chromosomes and detected the complete absence of the 3D chromosome (3D nullisomy) (Fig. 1b). When the mcGISH and FISH results were compared, the six wheat chromosomes with J hybridization signals were identified as the D-genome. The FISH probes also hybridized to alien chromosomes. All the *Thinopyrum* chromosomes had an Afa-family hybridization pattern in the telomeric region and three chromosomes had strong pTa71 signals in this region too. The centromeric and pericentromeric regions remained unlabelled, with only two chromosomes having Afa-family signals. A karyogram was constructed for the wheatgrass chromosomes present in this line and the FISH signals were summarized in an idiogram (Fig. 2a).

According to the mcGISH and FISH results the genome composition of line 194 is 14A+ 14B+ 12D + 8J + 8St + 2J^S^.

#### Partial amphiploid line 195: 56 chromosomes

**Fig. 3:**
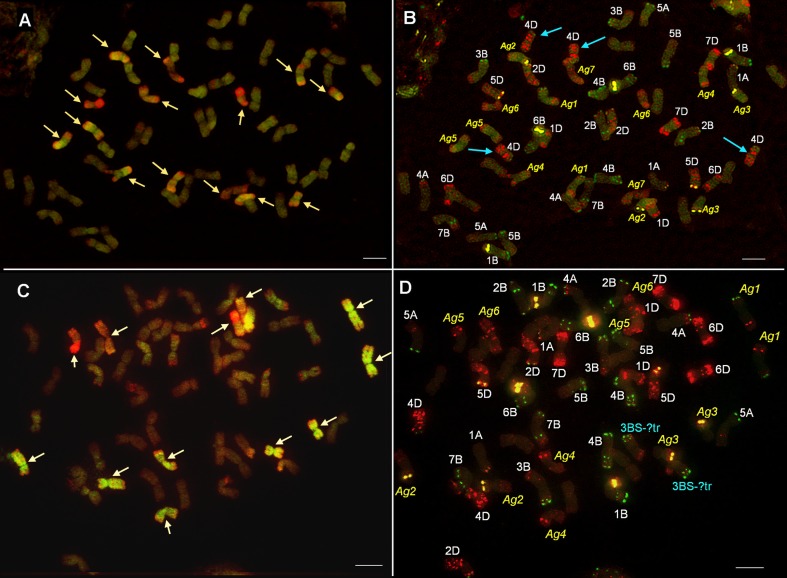
Multicolour genomic in situ hybridization (mcGISH) on mitotic chromosomes of the partial amphiploid lines 195 (**a**) and 196 (**c**) derived from the Mv9kr1 (wheat) × *Thinopyrum* synthetic hybrid (Agropyron glael, hybrid of *Thinopyrum intermedium* and *Thinopyrum ponticum*) cross using J (*Thinopyrum bessarabicum*, green) and St (*Pseudoroegneria spicata*, red) genomic DNA probes. Wheat chromosomes are unlabelled (brown). Alien chromosomes are indicated with arrowheads. The fluorescent in situ hybridization (FISH) pattern on the same cell of lines 195 (**b**) and 196 (**d**) using Afa-family (red), pSc119.2 (green) and pTa71 (yellow) repetitive DNA probes. A. glael chromosomes are numbered in yellow, not based on homology, while the wheat chromosomes are numbered in white. Four 4D chromosomes present in line 195 (**b**) are marked with blue arrowheads. Translocations between 3BS and an unidentified chromosome arm are marked with blue (**d**). Scale bar: 10 μm

Based on the mcGISH results seven pairs of chromosomes were identified as wheatgrass (Fig. 3a), five pairs of which seem to belong to the J genome as they were mainly green, and two pairs to the St genome, as they fluoresced red, though the hybridization pattern showed some specific features. A very bright red fluorescence signal was observed on the short arm of Ag6, while on the other arm the fluorescence was less intense. As the whole chromosome was red, it was classified as an St chromosome. Ag7 was also identified as an St chromosome, though the fluorescence signal was much fainter than in Ag6. A strong St genomic pattern was observed in the distal part of the short arms of the Ag1, Ag3 and Ag4 chromosomes, while other parts were green, which could be the result of a translocation between the J and St genomes.

The wheat chromosomes among which the D chromosomes exhibited slight fluorescence with the J genome probe were characterized using FISH. The chromosome-specific patterns identified two pairs of 4D and no 3D among the 42 wheat chromosomes, so this genotype was identified as a nullitetrasomic line (N3DT4D). During the FISH characterization of the *A. glael* chromosomes, only the Afa-family probe hybridized to Ag4, Ag5, Ag6 and Ag7, while a strong yellow pTa71 signal on Ag2 and Ag3 marked the NOR region of these chromosomes (Fig. 3b). A faint green pSc119.2 signal was visible in the distal part of the Ag1 short arm. A karyogram was constructed for the wheatgrass chromosomes present in this line and the FISH signals were summarized in an idiogram (Fig. 2b).

The chromosome composition of the progeny of the Mv9kr1/A. glael// Chinese Spring hybrid line 196 is 14A + 14B + 14D (nullitetrasomic line N3DT4D) + 10 J (including J-St translocations) + 4St

#### Partial amphiploid line 196: 54 chromosomes

McGISH discriminated six pairs of A. glael chromosomes, four pairs of which were hybridized strongly by J genomic DNA (Ag1-Ag4) over their entire length and exhibited great differences in chromosome length (Fig. 3c). The smallest J chromosome (Ag3) was nearly metacentric, while the others were acro- or telocentric. Digoxigenin-labelled St genomic DNA hybridized to the short arm of Ag5, while the long arm remained unlabelled. The last pair (Ag6) showed faint red fluorescence and was identified as St.

Twenty-one pairs of wheat chromosome were unlabelled, though the D chromosomes showed a low level of fluorescence intensity. FISH with repetitive DNA probes (Afa-family, pTa71, pSc119.2) was used for the identification of the 42 wheat chromosomes and showed the complete absence of the 3D chromosome (3D nullisomy) (Fig. 3d). This chromosome was substituted by another, which had 3BS as the longer arm and an unidentifiable small segment as the shorter arm. This small segment was not totally unlabelled by mcGISH, having weak green fluorescence like that observed for D genome-related chromosomes, suggesting the D or J genomic origin of the unknown segment. The FISH probes also hybridized to alien chromosomes. The Ag1, Ag4 and Ag5 chromosomes had Afa-family hybridization patterns in the subtelomeric region and two chromosomes (Ag2 and Ag3) had a strong pTa71 signal in the telomeric region. The centromeric and pericentromeric regions of the alien chromosomes remained unlabelled with the exception of Ag6, which had Afa-family signals. Probe pSc119.2 gave only a weak signal on the telomeric region of Ag1. A karyogram was constructed for the wheatgrass chromosomes present in this line and the FISH signals were summarized in an idiogram (Fig. 2c).

On the basis of the mcGISH and FISH results the genome composition of line 196 is 14A+ 14B + 2 3BS-D/J? translocation + 12D + 8J + 4St.

### Molecular marker analysis

The *STSLr19*_*130*_ marker gave PCR products of the expected 130 bp fragment size in the positive control wheat line S091-1027 and in the wheatgrasses *Th. intermedium*, *Th. ponticum* and A. glael. The primer pairs failed to amplify any fragments in the wheat parents Mv9kr1 and Chinese Spring and in the partial amphiploid lines, signalling the absence of *Lr19*.

**Fig. 4:**
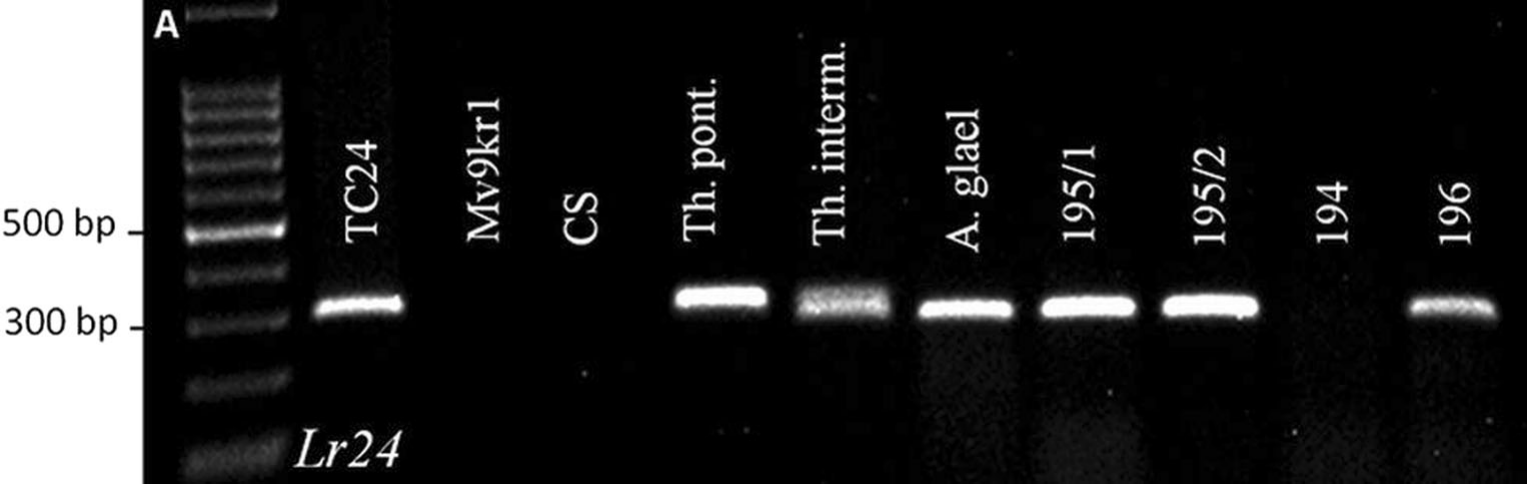
Agarose gel electrophoresis patterns of the J09-STS (*Lr24*) marker. The following DNA templates were used: positive controls (TC24); wheat genotype Mv9kr1, wheat cultivar Chinese Spring (CS), *Thinopyrum ponticum*, *Thinopyrum intermedium*, *Thinopyrum* synthetic hybrid (Agropyron glael, hybrid of *Thinopyrum intermedium* and *Thinopyrum ponticum*), Mv9kr1 wheat/Agropyron glael partial amphiploid lines 195 (two samples), 194 and 196. A 100-bp DNA ladder was used to estimate molecular weight

The *J09-STS* marker, which had complete linkage with *Lr24*, amplified the 310 bp fragment in the positive control wheat line TC24, in the wheatgrasses *Th. intermedium*, *Th. ponticum* and A. glael, and in the partial amphiploid lines 195 and 196. Line 194 showed no band intensity (Fig. 4).

With the help of the *Lr29*-linked Lr29F18-Lr29R18 primers, PCR products were identified in the TC29 positive control, *Th. intermedium* and *Th. ponticum*, while these primers gave no amplification products in A. glael, the wheat parents Mv9kr1 and Chinese Spring or the partial amphiploid lines.

The *Sr26#43* marker showed the presence of *Sr26* in the positive wheat control line Sunelg, *Th. ponticum* and A. glael, as PCR products were amplified at the expected 207 bp size. The *BE518379* marker showed band intensity at 303 bp size for the absence of *Sr26* in the wheat parents Mv9kr1 and Chinese Spring and the partial amphiploid lines.

### Phenotypic evaluation of the plants

**Fig. 5:**
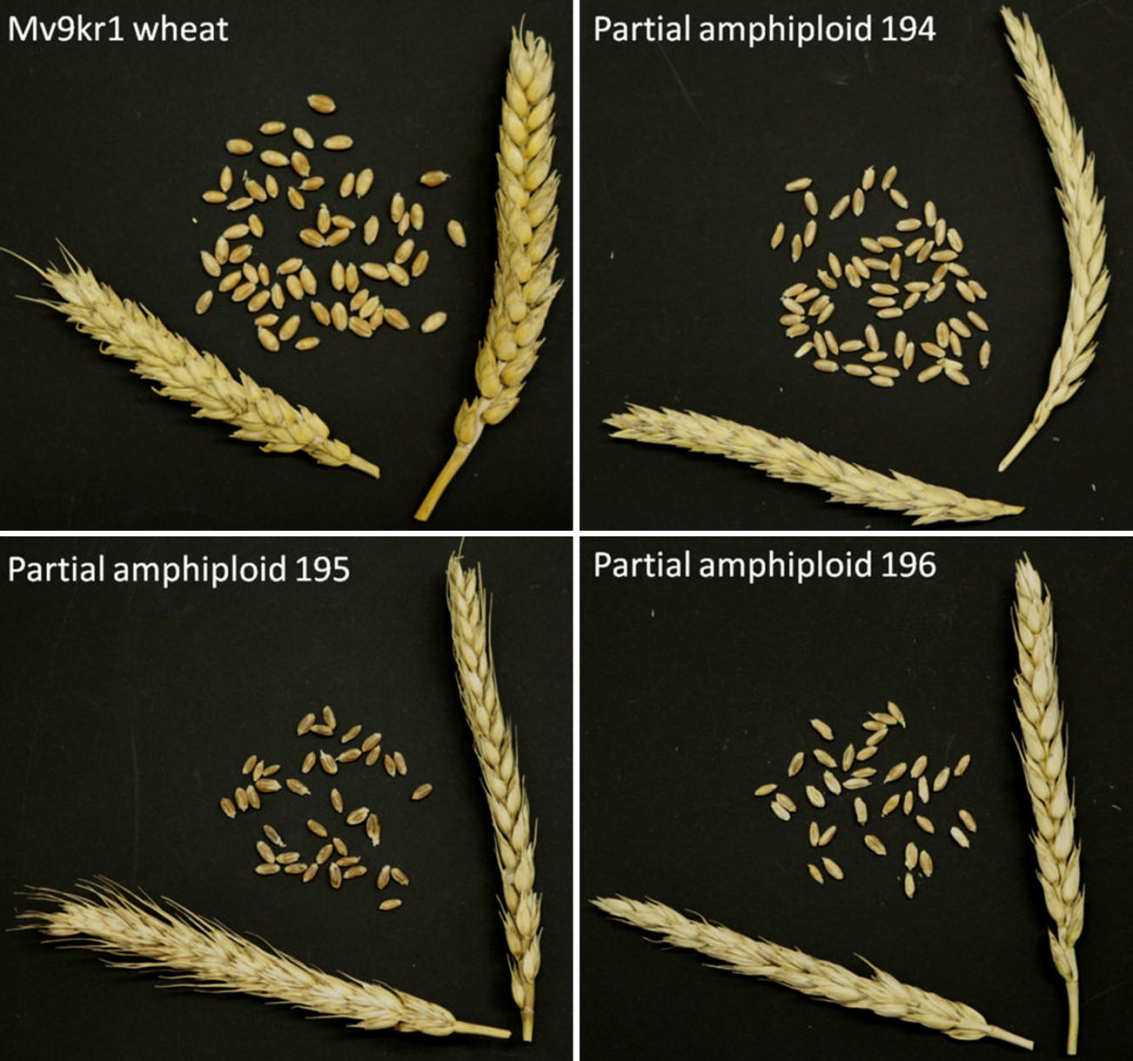
Spikes and seeds from a single spike of wheat genotpye Mv9kr1 and Mv9kr1/*Thinopyrum* synthetic hybrid (Agropyron glael, hybrid of *Thinopyrum intermedium* and *Thinopyrum ponticum*) partial amphiploid lines 194, 195 and 196. Martonvásár, Hungary, 2015

Phenotypically the partial amphiploids were closer to *Triticum aestivum*, whereas the adult plants expressed the characteristics of both parents. When the plants were evaluated in the field, the partial amphiploids were found to possess longer spikes (Fig. 5, Table 1) (10.1-13.2 cm) with good fertility (1.7-2.4 seeds/spikelet) and therefore produced no fewer kernels (39-55/spike) than the wheat parent (34-53/spike), except for dwarf line 196, which exhibited significantly lower fertility parameters in all the trials.

**Table 1:** Morphological traits of Mv9kr1/*Thinopyrum* synthetic hybrid (Agropyron glael) partial amphiploid lines (194, 195 and 196) grown in the field compared with the wheat parent Mv9kr1 (2014, 2015 Pesticide-free Tükrös nursery, Martonvásár; 2015 Breeder’s nursery, Lászlópuszta)

Year and location of field trials	Geno-type	Fertility (seeds/spikelet)	Plant height (cm)	Tillering (spikes/plant)	Length of main spike (cm)	Spikelets/main spike	Seeds/main spike
2014 Tükrös nursery	Mv9kr1	1.6 ± 0.1	99.0 ± 3.7	8.6 ± 2.2	10.2 ± 0.63	22.0 ± 1.1	34.4 ± 1.9
	L.194	1.8 ± 0.3	100.6 ± 6.8	5.9 ± 1.9*	12.4 ± 1.17*	25.4 ± 1.4*	44.6 ± 10*
	L.195	no data	no data	no data	no data	no data	no data
	L.196	1.5 ± 0.5	64.0 ± 6.8*	6.2 ± 2.2	13.2 ± 0.95*	24.0 ± 0.6*	36.4 ± 14.2
2015 Tükrös nursery	Mv9kr1	2.5 ± 0.3	68.9 ± 2.3	5.6 ± 2.2	8.4 ± 0.41	19.9 ± 1.5	50.1 ± 4.3
	L.194	2.1 ± 0.5*	100.0 ± 6.4*	5.5 ± 2.0	10.1 ± 0.88*	23.2 ± 2.4*	48.8 ± 13.0
	L.195	1.8 ± 0.5*	92.8 ± 8.9*	4.5 ± 1.6	12.0 ± 1.3*	21.7 ± 1.7*	39.1 ± 11.0
	L.196	1.1 ± 0.4*	57.2 ± 2.9*	5.0 ± 2.0	11.6 ± 1.65*	20.7 ± 2.3	22.1 ± 2.1*
2015 Breeder’s nursery	Mv9kr1	2.7 ± 0.2	75.3 ± 4.6	5.2 ± 0.8	9.1 ± 0.77	19.6 ± 2.5	53.8 ± 8.4
	L.194	2.4 ± 0.6	100.4 ± 4.8*	9.1 ± 2.9*	10.3 ± 0.97*	22.7 ± 1.9*	54.8 ± 14.2
	L.195	1.9 ± 0.3*	100.1 ± 5.2*	5.9 ± 2.1	13.2 ± 1.27*	25.3 ± 2.5*	48.2 ± 11.0
	L.196	1.8 ± 0.3*	69.6 ± 4.0*	7.8 ± 3.2*	11.3 ± 1.18*	22.6 ± 1.8*	40.0 ± 8.4*

*Significantly different from Mv9kr1 wheat at the P = 0.05 level

The seeds had characteristics intermediate between those of *Thinopyrum* and *T. aestivum*, as they were relatively thin and long, with darker brown colour and harder glumes than wheat. The flowering and harvesting times of the partial amphiploids were 10 to 15 days later in the field than for the wheat genotypes in all the years. All the partial amphiploids displayed a vigorous growth habit.

The results obtained for the MS Excel Student’s t-test can be found in Table 1. There were significant differences in morphological characters between the partial amphiploid lines and the control parental genotype Mv9krl. Line 194 had significantly longer spikes with more spikes per plant than the wheat parent in all the experiments, and the plants were significantly taller in both nurseries in 2015. When line 195 was evaluated in the Tükrös breeder’s nursery in 2015, the plant height, length of main spike and number of spikelets per main spike were found to be significantly higher than in wheat, but the fertility (number of seeds per spikelet) was lower. In the case of plant height and the length of the main spike the dwarf line 196 differed significantly from the wheat parental genotype Mv9kr1 in all the trials. The fertility and number of seeds/main spike were significantly lower than in wheat in two of the three experiments.

### Reaction to powdery mildew and rusts

**Fig. 6:**
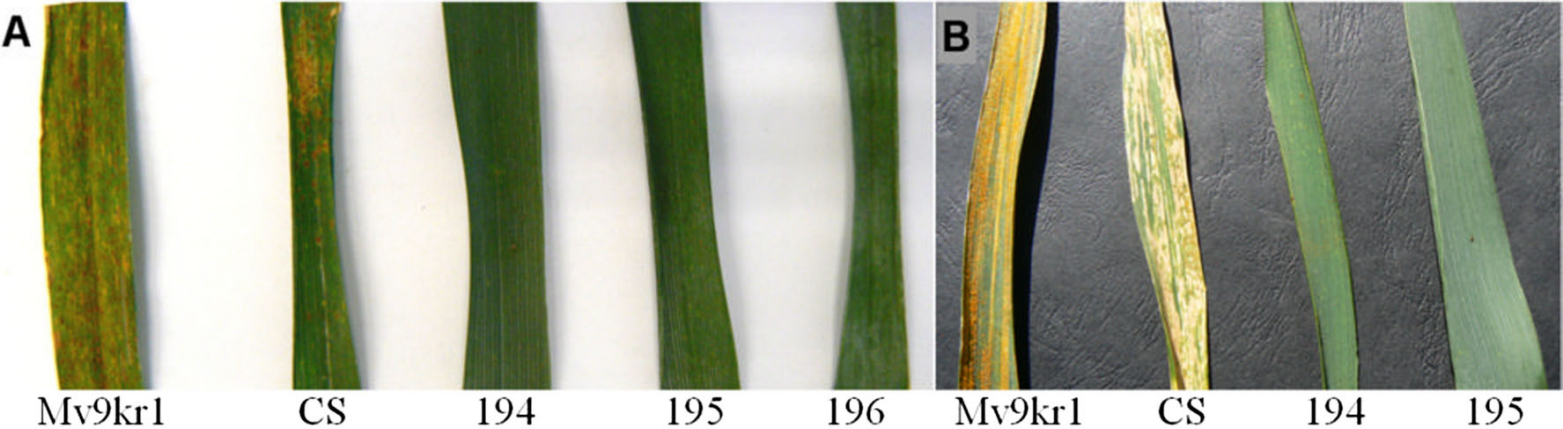
**a** Symptoms of spontaneous leaf rust infection on the flag-leaves of the susceptible wheat genotypes Mv9kr1 and Chinese Spring (CS) and of the leaf rust-resistant Mv9kr1/*Thinopyrum* synthetic hybrid (Agropyron glael, hybrid of *Thinopyrum intermedium* and *Thinopyrum ponticum*) partial amphiploid lines 194, 195 and 196. **b** Stripe rust infection on leaf of the susceptible wheat genotypes Mv9kr1 and Chinese Spring and healthy leaves of partial amphiploid lines 194 and 195. Pesticide-free nursery, Martonvásár, Hungary, 2014

Spontaneous leaf rust infection occurred in the pesticide-free Tükrös nursery in the years 2010–2015. During the developmental stage, the wheat–A. glael partial amphiploid lines were highly resistant (type 0) to the leaf rust isolates transmitted from the leaf rust-susceptible spreader rows in the Tükrös prebreeding nursery, while the wheat parents Mv9kr1 (type 4) and Chinese Spring (type 3) were heavily infected by the leaf rust pathogen in all five years (Fig. 6a).

Yellow rust infection was observed in 2014 and 2015 in the Tükrös nursery when, the disease occurred spontaneously. The Mv9kr1 and Chinese Spring cultivars were susceptible, while the partial amphiploids showed excellent resistance (Fig. 6b).

The partial amphiploids and their wheat parents were screened using isolates of powdery mildew. The three partial amphiploids and the wheat parents were highly susceptible (type 4) in the seedling stage.

## Discussion

In 2001 a crossing programme was begun using the wheat genotpye Mv9kr1 and A. glael (synthetic hybrid of *Th. intermedium* and *Th. ponticum*) wheatgrass in order to incorporate the disease resistance of A. glael into wheat (Molnár-Láng et al. 2012). The female wheat parent Mv9kr1 carried the *kr1* recessive gene, allowing high crossability in wheat × alien hybridizations (Molnár-Láng et al. 2010). As this wheat genotype is susceptible to leaf rust and yellow rust (Türkösi et al. 2014), the successful transfer of rust resistance from A. glael was easily recognizable in the hybrid progenies. As spontaneous leaf rust disease occurred in the nursery in Martonvásár in 2010–2015 (pesticide-free nursery, weather conditions conducive to fungi) there was no need for artificial inoculation. Partial amphiploid lines were selected from among the BC_1_ self-pollinated progenies. The aim of this work was to describe the chromosome composition and disease resistance of these unique lines by means of mcGISH and FISH and to compare their phenotypic components with those of the wheat parent Mv9kr1. Wheat/wheatgrass partial amphiploids originating from a cross with A. glael (*Th. intermedium* × *Th. ponticum* hybrid) have not previously been reported.

Chromosome counting on the partial amphiploids revealed 58 chromosomes (40 wheat + 18 alien) in line 194, 56 (42 wheat + 14 alien) in line 195 and 54 (42 wheat + 12 alien) in line 196. Other authors observed similar results in the case of wheat/*Th. intermedium* and wheat/*Th. ponticum* partial amphiploids. Most of the hexaploid wheat/*Thinopyrum* sp. partial amphiploids reported contained 56 chromosomes, consisting of 42 wheat and 14 *Thinopyrum* (Fedak et al. 2000; Han et al. 2004; Oliver et al. 2006; Georgieva et al. 2011; Bao et al. 2014; Zheng et al. 2014), though in some cases fewer wheat (38, 40) and more *Thinopyrum* chromosomes (16, 18) were observed (Chen et al. 1995; Fedak et al. 2000; Li et al. 2003; Yang et al. 2006; Oliver et al. 2006; Sepsi et al. 2008).

Wheat chromosome 3D was eliminated from the partial amphiploids. These BC_1_F_8_ lines were separated from each other in BC_1_F_3_, so the elimination of this chromosome probably happened earlier. Among the ABD genomes of hexaploid wheat, the D genome showed the closest homology to the J genome of *Thinopyrum* (Hsiao et al. 1995; Liu et al. 2007), which was confirmed by the more frequent presence of D-J substititutions and translocations than A-J or B-J (Qi et al. 2007). This close generic relationship could be observed during mcGISH, when J genomic probe DNA hybridized in some cases to D genome-related chromosomes. The hybridization pattern of St genomic DNA also had distinguishing features, as the NOR region of wheat chromosomes 1B and 6B and satellited wheatgrass chromosomes gave fluorescence signals with this probe in all cases. Tang et al. (2000) also described this phenomenon in *Th. intermedium*.

Decreased fluorescence intensity, J-St translocations in the telomeric region of J^s^ chromosomes, and unlabelled chromosome parts in all types of chromosomes were observed during mcGISH. Chen et al. (2001) reported a high frequency of chromosome pairing between J-J^S^, J-St and J^S^-St chromosomes, as the result of which genetic exchange is possible between these genomes. Several minor J-St and J^S^-St translocations were observed in the partial amphiploids. These translocations may have occurred during the formation of A. glael. As the J-J^S^-St chromosomes paired at high frequency, it may be that A. glael is not only a hybrid of the two wheatgrass species, that the genetic composition has changed or been enriched with DNA sequences from other species during the long maintenance period (decades), as wheatgrass species are open-pollinating and very polymorphic. As A. glael contains chromosomes from the two most valuable *Thinopyrum* species, changes in its genome could result in new invaluable genetic material for wheat breeding.

Among the wheatgrass chromosomes in the partial amphiploids, the FISH signals of those belonging to the J genome were compared to the FISH karyotype of the E genome (J^e^, E^e^ *, Th. elongatum*), published by Linc et al. (2012). Ag1 (lines 194, 195 and 196) was very similar to the 3E of *Thinopyrum elongatum*, while Ag2 (lines 194, 195, 196) was quite similar to 5E. Ag4 (line 194) showed the FISH pattern of 2E. The pTa71 FISH probe, which carries rDNA sequences, gave a strong hybridization signal in Ag2 and Ag3 in lines 194, 195 and 196, whereas Linc et al. (2012) only detected this on 5E. The pSc119.2 probe gave a signal on almost all the E chromosomes of the diploid *Th. elongatum* (Linc et al. 2012), but in the amphiploid lines only Ag1 (lines 194, 195, 196) and Ag5-Ag6 (line 194) hybridized with this probe. There were thus fewer FISH signals on the J chromosomes of A. glael than on diploid *Th. elongatum*. During allopolyploidization, rapid genomic events may eliminate non-coding, low-copy DNA sequences from homoeologous chromosomes, while reducing or amplifying high-copy DNA sequences, eliminating rRNA genes or repatterning chromosomes (Feldman and Levy 2005). When the FISH pattern of wheat and its progenitors *(T. urartu, Aegilops speltoides, Ae. tauschii*) were compared, a reduction in the number of FISH signals was also observed in wheat (Molnár et al. 2014).

Many wheat/*Th. intermedium* or wheat/*Th. ponticum* partial amphiploid lines have been reported to carry leaf rust resistance (Li et al. 2003; Han et al. 2004; Sepsi et al. 2008; Chang et al. 2010; Georgieva et al. 2011). The partial amphiploid lines identified in this study had excellent resistance to leaf rust, when observed over several years, but were susceptible to powdery mildew. In addition, the findings suggested that the partial amphiploids might carry different *Lr* and/or *Yr* genes, because they contained different types of wheatgrass chromosomes. The *Lr24* gene was detected in lines 195 and 196, but line 194 was also resistant to leaf rust. As the Mv9kr1 wheat parent is susceptible to leaf rust, it was concluded that the resistance of the three partial amphiploids originated from A. glael.

Phenotypically the partial amphiploids were similar to *T. aestivum*, but also expressed the characteristics of the wheatgrass parent and showed good viability. These lines were not just maintained in the nursery, but were used after successful propagation in new crossing programmes with modern, high-yielding wheat varietes in order to decrease the number of wheatgrass chromosomes and to incorporate leaf rust and yellow rust resistance through wheat-A. glael translocations. The selection and identification of resistant progenies is now in progress.
